# Once Daily Valacyclovir for Reducing Viral Shedding in Subjects Newly Diagnosed with Genital Herpes

**DOI:** 10.1155/2009/105376

**Published:** 2009-08-10

**Authors:** Mark G. Martens, Kenneth H. Fife, Peter A. Leone, Lynn P. Dix, Clare A. Brennan

**Affiliations:** ^1^Planned Parenthood of Arkansas and Eastern Oklahoma, Inc., 5780 S. Peoria Ave, Tulsa, OK 74105, USA; ^2^Department of Medicine, School of Medicine, Indiana University, Indianapolis, IN 46202, USA; ^3^Division of Infections Diseases, University of North Carolina, Chapel Hill, NC 27599, USA; ^4^GlaxoSmithKline, Research Triangle Park, NC 27709, USA

## Abstract

*Objective*. Genital herpes (GH) recurrences and viral shedding are more frequent in the first year after initial HSV-2 infection. The objective of this study was to provide the first evaluation of valacyclovir 1 g once daily compared to placebo in reducing viral shedding in subjects newly diagnosed with GH. *Methods*. 70 subjects were randomized to receive valacyclovir 1 g daily or placebo in a crossover design for 60 days with a 7-day washout period. A daily swab of the genital/anal-rectal area was self-collected for HSV-2 detection by PCR. Subjects attended the clinic for routine study visits and GH recurrence visits. Treatment differences were assessed using a nonparametric crossover analysis. *Results*. 52 subjects had at least one PCR measurement in both treatment periods and comprised the primary efficacy population. Valacyclovir significantly reduced HSV-2 shedding during all days compared to placebo (mean 2.9% versus 13.5% of all days (*P* < .01), a 78% reduction). Valacyclovir significantly reduced subclinical HSV-2 shedding during all days compared to placebo (mean 2.4% versus 11.0% of all days (*P* < .01), a 78% reduction). However, 79% of subjects had no GH recurrences while receiving valacyclovir compared to 52% of subjects receiving placebo (*P* < .01). *Conclusion*. In this study, the frequency of total and subclinical HSV-2 shedding was greater than reported in earlier studies involving subjects with a history of symptomatic genital recurrences. Our study is the first to demonstrate a significant reduction in viral shedding with valacyclovir 1 g daily compared to placebo in a population of subjects newly diagnosed with HSV-2 infection.

## 1. Introduction

Genital herpes is most commonly caused by infection with herpes simplex virus type 2 (HSV-2), and is one of the most prevalent sexually transmitted diseases (STD) worldwide [1–3]. The incidence of new infections is about 1 million cases per year in the United States [4]. At least 50 million persons in the United States have HSV-2 infection [5]. However, 86% of these individuals are unaware of their diagnosis, either because they do not have symptoms or because they have symptoms that go unrecognized [3, 6].

Previous studies demonstrated both viral shedding and recurrences are more frequent in the first year after initial HSV-2 infection [7–11]. In one natural history study, women who acquired genital herpes within a year before enrollment had more frequent shedding compared to women who had been infected for one year or more [8]. Another study found that individuals diagnosed for ≤6 months were twice as likely to shed asymptomatically compared to those diagnosed for more than 6 months [11]. Collectively, higher viral shedding and recurrence rates in subjects with newly acquired infection may translate to an increased risk of transmitting virus to uninfected sex partners.

In studies of immunocompetent adults with a known recurrence pattern, HSV-2 DNA as detected by polymerase chain reaction (PCR) testing was present on approximately 10% of days [12, 13]. Several investigations have supported the effect of suppressive antiviral therapy on the reduction of viral shedding in patients with an established recurrence pattern [9, 11, 14–17]. More recent studies have shown that suppressive antiviral therapy is as effective at controlling symptomatic disease in recently infected persons as it is in those with prevalent infection [18, 19]. Because clinical and subclinical reactivation of HSV may result in the sexual transmission of HSV to a partner, suppressing viral shedding is important at both the individual and population level [12]. The current study was conducted to provide additional natural history data in individuals who have been given a new diagnosis of genital herpes, and to provide the first evaluation of the efficacy of valacyclovir 1 g once daily in reducing HSV-2 shedding in this population. 

## 2. Materials and Methods

### 2.1. Design

This was a double-blind, randomized, multicenter, two-way crossover study conducted at 14 centers in the United States (US) between February and November 2006. Subjects were randomized to receive valacyclovir 1 g or matching placebo once daily for 60 days in each of two treatment periods in a two-way crossover design. In our study, the term VAL-PBO is used to describe the treatment order for subjects randomized to receive valacyclovir in the first treatment period followed by placebo in the second treatment period; the term PBO-VAL is used to describe the treatment order for subjects randomized to receive placebo in the first treatment period followed by valacyclovir in the second treatment period. There was a washout period of seven days between treatment periods. The 1 g dose of valacyclovir was selected to optimize treatment in this population, 20% of whom would be expected to have 10 or more outbreaks per year based on previous studies [7]. The primary endpoint was the percent of all days with any HSV-2 viral shedding over 60 days defined, for each subject as the percent of all days with PCR data for which HSV-2 shedding was detected by a positive PCR result. Secondary endpoints included percent of all days with subclinical shedding (without a genital lesion), percent of all days with clinical shedding (with a genital lesion), proportion of subjects with no shedding, proportion of subjects with at least one symptomatic recurrence and time to first symptomatic herpes recurrence.

### 2.2. Study Population

Eligible subjects were diagnosed with a first recognized episode of herpes at the screening visit or within 4 months of randomization. Subjects were required to have documented signs and symptoms of genital HSV-2 infection, as well as laboratory confirmation (positive culture, PCR, or type-specific serology) of HSV-2 infection to be eligible for the study. Subjects that were enrolled on the basis of a positive culture/PCR, but in the absence of HSV-2 antibodies, represented true incident HSV-2 infection. It was accepted that a subject's first recognized episode of genital herpes might not represent new infection as prior episodes may have escaped detection. All subjects signed an informed consent document approved by an appropriate review committee prior to any study procedures.

### 2.3. Procedures

A subject's participation in the study included a screening evaluation, a randomization visit, and eight study visits (four visits per treatment period, with a 15-day interval between each visit). HSV-1 and HSV-2 type-specific serologic testing was conducted at the screening visit. Immunocompromised subjects, and subjects seropositive for HIV, were excluded. Subjects were not tested for HIV infection at baseline. Once randomized, subjects attended the clinic for additional visits if they suspected they had a genital herpes recurrence. A genital examination was conducted at the screening visit for subjects who presented with genital herpes signs or symptoms. Genital examinations also were conducted at the randomization and genital herpes recurrence visits for all subjects. During each 60-day treatment period and during washout, subjects collected a single daily swab from their genital/anal-rectal area for HSV-2 detection by PCR. On Day 1 of a genital herpes recurrence, swabs of the lesion also were collected in the clinic for HSV-2 detection by culture and PCR. During a documented recurrence, a subject temporarily discontinued blinded study medication and received open-label episodic treatment with valacyclovir 500 mg twice daily for 3 days, after which double-blind therapy resumed. 

Subjects used a telephonic, interactive voice response system daily to capture information on genital lesions, genital symptoms, and oral outbreaks. Adverse events, concomitant medications, and treatment compliance information were collected at each clinic visit, based on recall and discussion with the subject as well as pill counts. Blood and urine samples were collected for hematology, clinical chemistry, and urinalysis at the screening visit and at the end of each treatment period. Pregnancy testing was performed at baseline and at the end of each treatment period.

### 2.4. Laboratory Methods

The HerpeSelect ELISA glycoprotein G-based assay (Focus Diagnostics, Cypress, Calif, USA) was used to detect the presence of HSV-1 and HSV-2 antibodies at screen (Quest Diagnostics, Van Nuys, Calif, USA). Samples demonstrating an index value >1.1 were considered positive. No confirmatory testing was performed. Viral isolates from cultures were confirmed and typed with monoclonal antibodies at Quest Diagnostics. HSV-2 DNA was assessed by qualitative florescent-based real time PCR assay at the University of Washington Virology Research Laboratory, Seattle, Wash, USA [20].

### 2.5. Statistical Analyses

In this study, it was estimated that 47 completed subjects provided at least 90% power to detect a reduction in the mean percent days of HSV-2 shedding from 9.6% in the placebo treatment phase to 2.8% in the valacyclovir treatment phase, assuming standard deviations of 9.8% and 5.6%, respectively, and a correlation of 0.4. This study also was powered for the secondary endpoint of subclinical shedding. Forty-seven completed subjects provided 90% power to detect a reduction in the mean percent days of subclinical HSV-2 shedding from 7.0% in the placebo treatment phase to 2.8% in the valacyclovir treatment phase, assuming standard deviations of 9.3% and 5.4%, respectively, and a correlation of 0.4. To allow for dropouts, a sample size of 66 subjects was chosen.

The populations considered in the overall analysis included the Intent-to-Treat (ITT) exposed, consisting of all subjects who received at least one dose of investigational product. This was the primary population for assessing safety. Also considered was the Intent-to-Treat Crossover (ITTC) population consisting of all ITT subjects who had at least one PCR result in each treatment period. This was the primary population for assessing efficacy. The First Period Efficacy population consisted of all patients with efficacy data in the ITT-Exposed, with the exception of 2 subjects whose data was compromised (see Section 3). This was the primary population for assessing efficacy in the first treatment period.

The treatment comparison for continuous endpoints, including the primary endpoint, was tested using nonparametric crossover analysis methods in the ITTC population [21]. The test of treatment difference was preceded by a test of carryover effect using a Wilcoxon rank-sum test at the 5% level of significance [21]. If this test was significant, then the treatment comparison would be tested using The First Period Efficacy population only. This analysis was carried out as a supporting analysis in any case because the test of carryover may have relatively low power.

The binomial secondary endpoints were analyzed using Prescott's method [22]. Time to first genital recurrence was evaluated with Kaplan-Meier estimates and log-rank test using the First Period Efficacy population and data in treatment period 1. The reported safety measures were adverse events and laboratory data.

## 3. Results

### 3.1. Subject Characteristics

Seventy subjects from 14 centers in the United States were randomized into the study, 35 subjects to the VAL-PBO treatment sequence, and 35 subjects to the PBO-VAL treatment sequence. During the course of routine monitoring, errors in sample identification were discovered for two subjects at one site. The error was felt to compromise the integrity of the data; therefore, swab samples from two subjects in the VAL-PBO sequence group were excluded from the efficacy analysis. An additional three subjects were “lost to follow-up” early in treatment period 1. These three subjects had some swabbing samples but all PCR assay results were missing, leaving 65 subjects in the First Period Efficacy population. 

Twenty of the 70 (29%) subjects in the ITT population prematurely withdrew from the study (11 while receiving valacyclovir, 9 while receiving placebo). The primary reason for withdrawal was “lost to followup” (*n* = 8, 11%), followed by “other reason” (*n* = 6, 9%), “subject decided to withdraw” (*n* = 4, 6%), and adverse event (*n* = 2, 3%). At least one major protocol deviation was recorded for 35 (50%) subjects randomized. Deviations included failure to take at least 80% of double-blind study medication (*n* = 13, 37%) and genital/anal-rectal swab PCR results on fewer than 85% of study days (*n* = 20, 57%).

The study population was predominantly female (70%); 51% were white (Table 1). Of the 70 subjects randomized, 9 (13%) were HSV-2 seronegative at entry indicating newly acquired HSV-2 infection. Note that one of these 9 subjects did not have efficacy data in treatment period 1. Of the 8 in the First Period Efficacy population, 6 were also HSV-1 negative, indicating primary infection. All nine subjects presented with laboratory confirmation of HSV-2 infection by culture or PCR at screen or upon initial diagnosis made within 4 months of randomization. These subjects represent a subset of the population who had a clearly documented recent HSV-2 infection.

### 3.2. Viral Shedding

The primary efficacy endpoint was the difference in mean percent of all days with HSV-2 shedding as determined by a type-specific HSV PCR assay. For the ITTC population, shedding was reported on a mean 13.5% of all days in subjects while receiving placebo compared to a mean 2.9% of days in subjects while receiving valacyclovir (*P* < .001), a significant decrease representing a 78% reduction in total HSV-2 shedding (Table 2). The test of period differences as well as the test of residuals (carryover effect) was not significant, *P* = .362 and *P* = .401, respectively. The supporting analysis of total days with shedding during treatment period 1 was consistent with the twoperiod carryover analysis. In the first treatment period, there was a statistically significant (83%) reduction in total days with shedding (Table 2).

The mean percent of all days with subclinical HSV-2 shedding was a secondary endpoint (Table 2). In the ITTC population, subclinical shedding was identified on a mean 11% of days in subjects while receiving placebo compared to a mean 2.4% of days in subjects while receiving valacyclovir (*P* < .001), a significant decrease representing a 78% reduction in subclinical shedding. Significant differences between valacyclovir and placebo also were demonstrated in the percent of days with clinical shedding, proportion of subjects with no shedding, proportion of subjects with no recurrences, and time to first recurrence (Table 2).

Valacyclovir 1 g once daily was well tolerated, with similar adverse event reporting compared to placebo. Two subjects withdrew from the study because of adverse events during treatment period 1 and did not progress to treatment period 2. One subject receiving placebo experienced a headache of moderate intensity; a second subject receiving valacyclovir experienced elevated alanine aminotransferase (ALT) and aspartate aminotransferase (AST) that were felt to be related to a concomitant medication. Both subjects withdrew without breaking the study blind and the relationship of the adverse event to the study drug was made while the drug was still blinded. 

**Figure 1:**
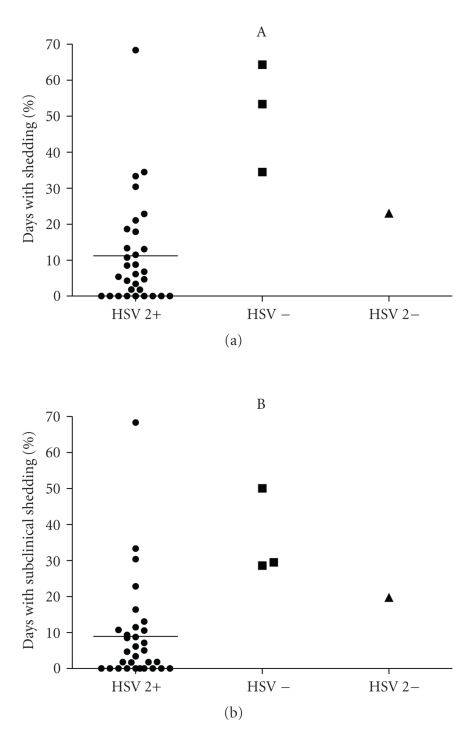
Scatter plot of shedding data for subjects receiving placebo in the first treatment period. The column labeled HSV-2 + shows data from subjects whose HSV-2 antibody test was positive at enrollment (*n* = 31). The horizontal line represents the mean value. The next column shows the individual values for the 3 subjects who were negative for both HSV-1 and HSV-2 antibody; the next column shows the one subject who was HSV-1 antibody positive and HSV2 antibody negative at enrollment. (a) Total HSV-2 shedding, and (b) subclinical shedding.

A post-hoc analysis of the 8 subjects with documented incident HSV-2 infection was conducted to explore shedding and recurrence rates in this subset of participants. In an analysis of first period placebo treatment data, 4 subjects were HSV-2 seronegative including 3 who were also HSV-1 seronegative (primary infection) and 31 subjects were HSV-2 seropositive. This analysis suggests that recently infected subjects in the first period placebo group shed HSV-2 more often than those already with HSV-2 antibody (Table 3  and Figure 1). In an analysis of first period valacyclovir treatment data, 4 subjects were HSV-2 seronegative (including 3 who were also HSV-1 seronegative), and 26 subjects were HSV-2 seropositive. Among subjects who received valacyclovir in the first treatment period, similar results were obtained for HSV-2 seronegative and seropositive subjects with respect to total days with shedding (5.9% versus 2.1%) and the proportion of subjects recurrence-free (100% versus 81%). The percentage of days with subclinical shedding was higher in the HSV-2 seronegative group compared to the HSV-2 seropositive (5.9% versus 1.4%, *P* = .05); however, the study was not powered for this comparison.

## 4. Discussion

The present study is the first to evaluate valacyclovir 1 g once daily to reduce HSV-2 viral shedding in a population of subjects newly diagnosed with genital herpes infection. In our study, valacyclovir 1 g once daily significantly reduced viral shedding by 78% compared to placebo. The effect of suppressive valacyclovir therapy on reducing viral shedding and reducing the frequency of genital recurrences is similar to that reported in earlier studies that enrolled persons with an established history of genital herpes [12, 13, 18, 19]. 

Results from a large, randomized, controlled study demonstrated that valacyclovir suppression (500 mg daily) reduced transmission of symptomatic HSV2 infection by 75% and overall acquisition (HSV-2 seroconversion) by 48% in discordant couples [12]. Within this trial, a viral shedding substudy of 89 patients evaluated for 60 days demonstrated a 73% reduction in total days of viral shedding (mean 2.9% versus 10.8%, as measured by PCR) and a 64% reduction in days of subclinical viral shedding (mean 2.8% versus 7.8%) in infected source partners receiving valacyclovir compared to those receiving placebo.

In a study of valacyclovir 1 g once daily for the reduction of HSV-2 viral shedding in 152 subjects with a history of ≥6 genital herpes recurrences per year, the mean percent of days with HSV-2 shedding was 9.3% in the placebo group and 2.7% in the valacyclovir group, a 71% reduction (*P* < .001). The mean percent of subclinical days with shedding was 6.4% on placebo and 2.7% in the valacyclovir group, a 58% reduction (*P* < .001) [13].

In another HSV-2 viral shedding study that enrolled a heterogeneous population of 69 subjects (newly diagnosed with HSV-2 infection, and diagnosed >6 months prior to study entry), participants received oral valacyclovir 500 mg BID, acyclovir 400 mg BID, and placebo in random order for 7 weeks each, in a three-period crossover design [11]. There was a one-week washout between treatment periods. However, 27 subjects (39%) had a diagnosis of a first episode of genital herpes ≤6 months before randomization. Overall, for the 69 subjects randomized, HSV DNA was detected by PCR from at least one anatomical site on 40.2% of the days while receiving placebo, compared to 8.0% of days while receiving acyclovir (80% reduction) and 7.2% of days while receiving valacyclovir (82% reduction).

In the current study, the mean percent of days with total HSV-2 shedding was 2.9% while receiving valacyclovir, compared to 13.5% while receiving placebo. These results are similar to those of the first two studies [12, 13] noted above except that the proportion of days with shedding while on placebo was slightly higher in the current study. It is not clear if that difference is meaningful, but it could be because our subjects were recently infected and these persons shed virus more frequently than those with established disease [8, 9]. The study by Gupta et al. reported rates of shedding in both the placebo and treatment phases that were higher than those of the current study. However, the proportional reduction of shedding in subjects on suppression in our study was similar to that reported by Gupta et al. The reason for the discrepancy between the results reported by Gupta et al. and other studies, including the one reported here, is uncertain, but could be related to the multiple swab technique used in the earlier study or in the selection of study subjects [11].

Of the 70 subjects in this study, 9 (13%) were HSV-2 seronegative at the time of diagnosis by culture or PCR. These subjects clearly had new HSV-2 infections while the remaining study subjects had been infected long enough to mount an antibody response by the time they enrolled in the study. This provided us with the opportunity to explore the differences in viral shedding between those with and without antibodies to HSV-2 in the group randomized to placebo treatment in the first study period. As was previously reported in a study that evaluated viral shedding by culture in initially HSV-2 seronegative subjects [23], asymptomatic shedding was extremely common. Our post-hoc analysis showed shedding rates 4-to5-fold higher among those initially HSV-2 seronegative subjects compared to the broader group of subjects who were newly diagnosed but already HSV-2 seropositive. Given the limited number of patients in the HSV-2 seronegative subset, the viral shedding results in this cohort must be interpreted with caution, yet the observation suggests the potential importance of early treatment in persons with new HSV-2 infection. 

The CDC advocates the use of suppressive therapy for reducing genital herpes recurrences in patients with frequent recurrences (≥6 recurrences/year), and states treatment also is effective in patients with less frequent recurrences (5). In addition, sexual transmission of HSV can occur during asymptomatic periods, and asymptomatic viral shedding is most frequent during the first 12 months after acquiring HSV-2 (5). Two recent studies have shown that suppressive therapy with valacyclovir in subjects with newly diagnosed HSV-2 genital herpes has clinical efficacy in reducing outbreaks, similar to that seen in persons with established recurrent disease [18, 19]. The current study extends those findings by showing that the impact of suppressive valacyclovir on viral shedding is also similar to that found in persons with established recurrent genital herpes. Taken together, these studies suggest that there is no reason to wait until a person with newly diagnosed genital herpes establishes a recurrence pattern to consider suppressive therapy. In fact, because of the high rate of viral shedding and high probability of clinical recurrences, persons newly diagnosed with HSV-2 infection may be among the best candidates for suppressive therapy.

## Figures and Tables

**Table 1:** Demographics ITT Population.

Demographics	VAL-Placebo	Placebo-VAL
No. of subjects	35	35
Female gender, *n* (%)	22 (63)	27 (77)
Mean Age, years (SD), range	29 (9), 18–49	33 (10), 20–58
White, *n* (%)	19 (54)	17 (49)
Black, *n* (%)	13 (37)	15 (43)
HSV-1^a^ and HSV-2 Antibody Positive, *n* (%)	17 (49)	19 (54)
HSV-1 Antibody Positive, HSV-2 Antibody Negative, *n* (%)	2 (6)	1 (3)
HSV-1 Antibody Negative, HSV-2 Antibody Positive, *n* (%)	13 (37)	12 (34)
HSV-1 and HSV-2 Antibody Negative, *n* (%)	3 (9)	3 (9)

^a^One patient in each treatment group had an HSV-1 equivocal result and are included here.

**Table 2:** Percent of Days with HSV-2 Viral Shedding.

Population ITTC		Valacyclovir	Placebo	*P*-value	% Reduction
		(*n* = 52)	(*n* = 52)		
Period 1	*n*	25	27		
	Mean (SD %)	3.0 (4.6)	14.3 (16.6)		
	Range	0–17	0–68		

Period 2	*n*	27	25		
	Mean (SD %)	2.9 (6.5)	12.6 (17.4)		
	Range	0–29	0–61		

Overall	*n*	52	52		
	Mean (SD %)	2.9 (5.6)	13.5 (16.9)	<.001	78
	Range	0–29	0–68		

ITT Period 1	*n*	30	35	<.001	83
	Mean (SD %)	2.6 (4.3)	14.9 (18.3)		

Other Efficacy Parameters					

ITTC		Valacyclovir (*n* = 52)	Placebo (*n* = 52)	*P*-value	

Percent days with subclinical shedding, Mean (SD %)		2.4 (4.8)	11 (15.1)	<.001	
Percent days with clinical shedding, Mean (SD %)		0.6 (1.7)	2.4 (4.4)	.014	
Subjects with no Shedding, *n* (%)		31 (60)	15 (29)	<.001	
Proportion of subjects recurrencefree, *n* (%)		41 (79)	27 (52)	.003	
Median time to first genital herpes recurrence^a^ (days)		>68	61	.010	

^a^First treatment period.

**Table 3:** Post-hoc Analysis of Patients with Documented Incident Infection—Results from the First Treatment Period.

	HSV-2 Antibody	HSV-2 Antibody
	Negative ^a^ *n* = 4	Positive *n* = 31
Percent days with total shedding, Mean (SD %) *P*-value^b^	43.8 (18.6 )	11.2 (14.8)
	*P* < .001	
		

Percent days with subclinical shedding, Mean (SD %) *P*-value^b^	31.9 (12.8 )	8.9 (14.1)
	*P* = .002	
		

Proportion of subjects recurrence-free^c^, *n* (%)	0 (0)	19 (61)

^a^This group includes 3 subjects who were HSV-1 and HSV-2 seronegative, and one subject who was HSV-1 seropositive and HSV-2 seronegative. ^b^This was an exploratory analysis; *P*-values should be regarded only as a rough guide to the magnitude of differences between groups. ^c^This difference was statistically significant (*P* = .04); however, this was an exploratory analysis.
